# Barriers, benefits, and unmet needs in full-time, off-the-job training among oncology nurses: a qualitative descriptive study

**DOI:** 10.3389/fmed.2026.1979432

**Published:** 2026-10-08

**Authors:** Bohui Shi, Yanqing Kang, Wenqian Zhao, Ya Zhang, Bin Wang, Ziling Chang, Huifang Zhang, Xiaona Liu, Jing Wang, Jieqiong Li

**Affiliations:** 1 Department of Breast Surgery, The First Affiliated Hospital of Xi’an Jiaotong University, Xi’an, Shaanxi, China; 2 School of Nursing, Sun Yat-sen University, Guangzhou, Guangdong, China; 3 The Nethersole School of Nursing, Faculty of Medicine, The Chinese University of Hong Kong, Hong Kong, Hong Kong SAR, China; 4 School of Nursing, Health Science Center, Xi'an Jiaotong University, Xi'an, Shaanxi, China; 5 Department of Nursing, The First Affiliated Hospital of Xi’an Jiaotong University, Xi’an, Shaanxi, China

**Keywords:** continuing professional development, off-the-job training, oncology nurses, qualitative descriptive study, specialty nursing

## Abstract

**Background:**

Full-time, off-the-job training is widely used to enhance clinical competency and support the continuing professional development of nursing staff. In oncology settings, however, there remains a paucity of evidence regarding how nurses experience this training and what barriers and unmet needs influence its impact upon their return to clinical practice. Hence, this study explores the perceived benefits, barriers, and unmet needs associated with full-time, off-the-job training among oncology nurses.

**Methods:**

A qualitative descriptive design was employed. Semistructured, in-depth interviews were conducted with 15 nurses from oncology-related departments at a large tertiary hospital. Participants had attended full-time, off-the-job training within the previous 5 years. Interviews were audio-recorded, transcribed verbatim, and analyzed using conventional qualitative content analysis. Data were collected between December 2023 and May 2024.

**Results:**

Our study revealed that participants viewed off-the-job training as essential to maintaining specialty competence in oncology nursing practice. Importantly, the training generated significant professional and interpersonal benefits, including the acquisition of specialized knowledge, the expansion of professional networks, and increased career motivation. Moreover, participation was shaped by systemic barriers, including limited departmental support, work-family conflict, and a lack of institutional recognition or role adjustment following training. Finally, nurses reported unmet needs in training design and post-training integration, including stronger alignment with clinical practice, more flexible delivery, better feedback, opportunities to apply newly acquired competencies, and clearer career pathways.

**Conclusions:**

The efficacy of off-the-job training depends not only on the availability of educational opportunities but also on robust organizational support, training relevance, and clear pathways for role integration and career advancement.

## 1 Introduction

Oncology nursing practice is characterized by increasing complexity driven by continuing advancements in cancer therapeutics, symptom management, supportive care, vascular access, and health informatics (1, 2). As patient needs become more complex and care pathways more specialized, oncology nurses are expected not only to provide safe and effective bedside care but also to continuously update specialty knowledge, refine clinical judgment, and respond to rapidly changing practice demands (1, 3). Strengthening specialty competence among oncology nurses is therefore essential for maintaining the quality of care and supporting the long-term development of the oncology nursing workforce (3, 4).

Full-time off-the-job training is one strategy commonly used to support nurses' continuing professional development (5, 6). In Chinese tertiary hospitals, such oncology nursing training usually spans 2–6 consecutive months, with trainees temporarily relieved from routine clinical work (7). By stepping away from routine work responsibilities for a defined period, nurses may gain opportunities for concentrated learning, skills development, professional exchange, and broader career planning. In principle, such training is particularly significant within oncology settings, where nursing staff must adapt to evolving standards, increasingly specialized procedures, and multidisciplinary models of care (8). Prior work has also suggested that effective oncology professional development extends beyond technical updating alone and includes structures such as mentorship, sponsorship, feedback, and career planning (9).

However, the value of training depends on more than access to training opportunities alone. Nurses may encounter competing work and family responsibilities, limited departmental support for release from clinical duties, and uncertainty about how newly acquired competencies can be applied after returning to practice (10, 11). In addition, training programs often lack alignment with specific clinical requirements and fail to provide formalized trajectories for post-training professional development (12). Existing evidence suggests that oncology nurses continue to report unmet training needs, and that institutional policies and structures are important in determining whether training translates into role performance and patient-centered care (13). Similar gaps in organizational support and training transfer for oncology nurses have also been identified across several Asian countries (2). Broader work on the oncology nursing workforce has similarly emphasized the importance of continuing specialized education, organizational support, and visible career pathways for professional development and retention (4). These challenges vary in smaller hospitals, where shortages of specialist training resources and limited access to off-the-job training opportunities are more prevalent (7, 14). In contrast, tertiary hospitals have relatively accessible training programs but face organizational barriers to releasing nurses from clinical duties and sustaining post-training competency application.

In China, oncology nursing has developed rapidly in recent years, yet challenges remain in specialty training, post-training role utilization, and the standardized development of oncology nurses (3, 7, 14, 15). These issues may be especially salient in large tertiary hospitals, where nurses are expected to deliver increasingly specialized care while also navigating complex organizational structures and workforce pressures. Limited evidence regarding the training experiences and systemic barriers of Chinese oncology nurses remains a critical gap; without robust clinical integration and organizational support, such programs yield suboptimal impacts on care quality and workforce retention (4, 9, 13).

Therefore, this study aimed to explore the barriers, benefits, and unmet needs of full-time, off-the-job training among oncology nurses in a tertiary hospital in China. By examining how nurses experience participation in off-the-job training and how they perceive its value, constraints, and post-training implications, this study seeks to inform the design of more responsive training programs and to provide practical implications for nursing management, specialty workforce development, and post-training role integration.

## 2 Methods

### 2.1 Design

Adopting a qualitative descriptive design, this study explored the barriers, benefits, and unmet needs of full-time training for oncology nurses. While no formal, predefined theoretical or conceptual framework was used to define coding categories or constrain theme generation during study conceptualization, interview guideline development, or data analysis, several sensitizing interpretive lenses drawn from the existing literature informed the inquiry. These include the Baldwin-Ford training-transfer model, concepts of continuing professional development in nursing, organizational support for specialist nurses, and specialist-nurse role development. These perspectives acted as broad interpretive guides rather than deductive templates; all themes emerged inductively from participant narratives. This approach provides a comprehensive, low-inference summary of participant experiences while remaining close to the original data (16–18). This study is reported in accordance with the Consolidated Criteria for Reporting Qualitative Research (COREQ) (19).

### 2.2 Setting and participants

Participants were recruited from oncology-related departments of a large tertiary hospital in Shaanxi Province, China, including medical oncology, radiotherapy, breast surgery, general surgery, thoracic oncology surgery, and abdominal oncology surgery. Eligible participants were nurses engaged in clinical nursing practice in oncology-related departments who had attended full-time, off-the-job training within the previous 5 years and were willing to participate in an interview. Nurses were excluded if they had participated in similar research or possessed less than 3 years of clinical experience. This 3-year clinical experience threshold was set by the hospital's local admission rule for its full-time, off-the-job oncology specialist training: nurses with fewer than 3 years of clinical practice are generally ineligible to enroll in the training program. Eligible participants were approached primarily face-to-face in their departments. One participant was interviewed by telephone because an in-person interview was not feasible. No invited participants refused to participate or withdrew from the study. When some nurses were unavailable at the initial invitation, the interview was rescheduled at a time convenient for them.

### 2.3 Sampling

Purposive maximum-variation sampling was used to capture a range of perspectives across departments, professional titles, educational backgrounds, and years of clinical experience. Fifteen nurses were ultimately interviewed. Sampling continued until informational sufficiency was reached and no new themes or subthemes emerged from the interviews.

### 2.4 Data collection

Data were collected between December 2023 and May 2024. A semistructured interview guide was developed by the research team after a review of the literature. The initial guide was designed by the first author, who had a solid background in oncology nursing and prior training in qualitative interviewing, and was further discussed and revised by the research team. The guide was refined after pilot interviews and finalized before formal data collection (Table 1). The guide explored participants' overall training experiences, perceived meaning of training, motivations for participation, barriers and facilitators during training, preferred training characteristics, perceived benefits, post-training application, support needs, and suggestions for improvement.

**Table 1:** Semistructured interview guide.

No.	Interview question
1	Please describe your overall experience of participating in full-time, off-the-job training as an oncology nurse.
2	What did full-time, off-the-job training mean to you in relation to your specialty practice?
3	Why did you decide to participate in the training?
4	During the training process, what factors helped you continue participating, and what difficulties or burdens did you encounter?
5	Which aspects of the training did you find most useful, and which aspects did not meet your needs?
6	What benefits do you think this training provided for your professional practice and development?
7	After returning to work, how were you able—or unable—to apply what you had learned in practice?
8	What changes or support do you think are needed to improve future full-time, off-the-job training programs for oncology nurses?

Data collection was conducted by 2 members of the research team with training in qualitative interviewing and experience in oncology nursing. During interviews, the interviewers remained neutral and nonjudgmental and encouraged participants to describe their experiences in detail. One interviewer led the interview, while the other documented nonverbal information, including tone, facial expression, pauses, and emotional reactions. Interviews were conducted in a private office, audio-recorded with participants' consent, and lasted approximately 30–60 min. Audio recordings were transcribed verbatim within 24 h after each interview. No non-participants were present during the interviews. Formal repeat interviews were not conducted; however, when clarification was needed during data organization, participants were re-contacted to confirm specific points.

### 2.5 Data analysis

Data were managed using NVivo 11.0 and analyzed through conventional qualitative content analysis (16). This inductive analytical approach did not rely on a predefined theoretical or conceptual framework for deductive coding, although sensitizing concepts from the training-transfer and continuing-professional-development literature supported *post hoc* interpretive sense-making, allowing categories and themes to emerge naturally from participants' narratives (20). The analysis followed a five-step iterative process. First, transcripts were read repeatedly to obtain an overall understanding of the data. Second, meaning units relevant to the study aim were identified and coded line by line. Third, transcripts and initial coding were checked against the audio recordings by 2 members of the research team to reduce transcription and coding bias. Fourth, the preliminary codes were organized into categories, subthemes, and broader themes. Formal analysis and theme development were led by one member of the research team with support from the first author, while the corresponding author supervised and reviewed the analytic process. Fifth, key analytic decisions, including sampling, interview guide development, coding, category refinement, and theme generation, were discussed within the research team until consensus was reached. The analysis moved iteratively between transcripts, codes, subthemes, and themes until no new themes or subthemes emerged.

### 2.6 Ethical considerations

This study was conducted in line with the Declaration of Helsinki and approved by the Ethics Committee of the First Affiliated Hospital of Xi'an Jiaotong University (No. XJTU1AF2023LSK-489). All participants were informed of the study purpose, the potential risks and benefits of participation, their right to withdraw at any time, and the measures taken to protect confidentiality. Written informed consent was obtained from all participants before data collection.

### 2.7 Methodological rigor

Several strategies were used to enhance the methodological rigor of the study. Interviewers had prior training in qualitative research and interviewing and used a semistructured interview guide that was developed, discussed, pilot-tested, and refined by the research team. Verbatim transcripts were completed within 24 h after each interview and checked against the audio recordings by 2 members of the research team to enhance accuracy. Two researchers independently performed line-by-line inductive coding based on raw interview narratives, rather than adopting a template-based coding framework. Rather than calculating numerical intercoder reliability, coding discrepancies were addressed via iterative team discussion. When uncertainties arose during data organization, participants were re-contacted to verify specific details. After independent coding and consensus integration, a formal codebook with standardized code definitions and representative exemplar quotes was compiled to ensure analytical transparency and consistency. Preliminary interpretations of the findings were also shared with several participants to assess whether the themes were consistent with their experiences. In addition, the research team discussed data coding, category development, and theme generation at key analytic stages, and disagreements in interpretation were resolved through consensus. An audit trail of key methodological and analytic decisions was maintained throughout the research process, and purposive maximum-variation sampling supported transferability.

### 2.8 Researcher reflexivity

Given the research team's familiarity with the clinical context, proactive measures were taken to monitor the potential influence of prior knowledge on data generation and interpretation. Importantly, the interviewers were not in direct managerial roles over the participants, which helped reduce the potential influence of hierarchical relationships. Throughout data collection and analysis, the research team discussed assumptions, reflected on possible bias, and sought to remain grounded in participants' accounts rather than prior expectations. The interviewers were female nurses with prior clinical experience in oncology nursing and training in qualitative interviewing.

## 3 Results

### 3.1 Participant characteristics

A total of 15 nurses (13 female, 2 male) from 6 oncology-related departments participated in the study (Table 2), including medical oncology, abdominal oncology surgery, thoracic oncology surgery, breast surgery, radiotherapy, and general surgery. Professional titles included senior (*n* = 3), intermediate (*n* = 9), and junior (*n* = 3) levels. Interviews lasted a total of 482 min and generated more than 80,000 words of transcript. Data analysis yielded 135 initial codes, 13 subthemes, and 5 overarching themes. Themes, sub-themes, brief descriptions, and representative codes are summarized in Supplementary Table S1.

**Table 2:** Participant characteristics (*N* = 15).

Participant	Age range, y	Education level	Professional title	Department
P1	35–44	Undergraduate	Intermediate	Medical oncology
P2	45–54	Graduate	Senior	Medical oncology
P3	25–34	Undergraduate	Junior	Abdominal oncology surgery
P4	35–44	Undergraduate	Intermediate	Breast surgery
P5	35–44	Undergraduate	Intermediate	Radiotherapy
P6	35–44	Undergraduate	Intermediate	Thoracic oncology surgery
P7	35–44	Undergraduate	Intermediate	Radiotherapy
P8	25–34	Undergraduate	Junior	Thoracic oncology surgery
P9	35–44	Undergraduate	Intermediate	General surgery
P10	55–64	Undergraduate	Senior	Breast surgery
P11	35–44	Graduate	Senior	Abdominal oncology surgery
P12	25–34	Graduate	Intermediate	General surgery
P13	25–34	Undergraduate	Junior	Radiotherapy
P14	25–34	Undergraduate	Intermediate	Abdominal oncology surgery
P15	35–44	Undergraduate	Intermediate	Thoracic oncology surgery

Age is presented in ranges to reduce identifiability in a single-hospital sample. Education level is grouped as undergraduate or graduate to further protect participant anonymity. Professional titles reflect the Chinese title system and are presented as junior, intermediate, and senior.

### 3.2 Thematic findings

Analysis of the interview data identified 5 major themes and 13 subthemes: (I) perceived necessity of off-the-job training for specialty oncology practice, (II) multidimensional value of training, (III) competing organizational, personal, and role-related burdens, (IV) misalignment in training design, delivery, and feedback, and (V) expectations for post-training application and career pathways.

#### 3.2.1 Theme 1: perceived necessity of off-the-job training for specialty oncology practice

##### 3.2.1.1 Subtheme 1: specialty practice demands

Participants consistently viewed full-time, off-the-job training as essential rather than optional. They described oncology nursing as a rapidly evolving specialty in which nurses are expected to deliver not only routine bedside care but also increasingly specialized, professional, and comprehensive services. Several participants suggested that without adequate training, nurses would struggle to meet patient expectations and maintain specialty credibility. As one participant noted, “If our own specialty competence is not strong enough, patients may feel that there are big differences between departments. For procedures such as PICC, implantable ports, or midlines, patients often know a great deal themselves. If we are not standardized and consistent, they will think we are not professional” (P5). Another participant added, “The expectations for nurses are now really high. It is no longer just about clinical nursing, but also teaching, research, and all-around development” (P10).

##### 3.2.1.2 Subtheme 2: clinical and technological updating

Participants also emphasized that off-the-job training was necessary to keep pace with continuing advances in oncology-related knowledge and procedures. They described training as a means of avoiding professional stagnation and staying current with changing guidelines, expert consensus statements, and specialty operations. One participant explained, “Training is really necessary. Only by going out to learn or by learning from other departments can you know what others are doing. Otherwise, you may just stay in your own small corner. Your vision and perspective can change a lot through learning” (P5). Another said, “Knowledge is being updated very quickly now. Guidelines and expert consensus can change at any time. If you do not keep learning, even after a short while, things may already be different” (P7). A third participant highlighted the same issue in relation to procedures: “For operation-related training, such as implantable ports, PICC maintenance, and ostomy care, there are updates every year. Sometimes there are more refined and better ways of doing things, so it is impossible to learn once and continue in the same way forever. Ongoing training is necessary” (P3).

#### 3.2.2 Theme 2: multidimensional value of training

Participants described off-the-job training as generating value across multiple domains. In addition to improving specialty knowledge and clinical competence, they emphasized the interpersonal and career-related benefits of training. Training was therefore experienced not simply as an educational activity, but as a broader developmental opportunity.

##### 3.2.2.1 Subtheme 1: professional competence

At the professional level, participants reported that training improved their specialty knowledge, technical skills, and clinical thinking. They valued opportunities to deepen their understanding of oncology nursing concepts and emerging treatment approaches, and they believed training strengthened their ability to respond to complex clinical problems. One participant described the impact of specialty expertise by referring to a wound care nurse in her department: “Some patients have wound-related problems, and after our wound specialist nurse helped them solve these problems, they were extremely appreciative. Sometimes they even said that doctors could not solve the problem, but Nurse Yang did, and they all said she was very professional” (P3). Another participant stated, “Relevant training can improve both theoretical and technical competence, and it can also improve evidence-based thinking at work. This is what clinical nurses lack the most. With this way of thinking, many clinical problems can be solved much more easily” (P2). Similarly, one participant noted, “Some new treatment methods are not covered sufficiently, comprehensively, or specifically in textbooks. Training is needed to make up for this knowledge gap so that we can improve our ability to deal with clinical problems” (P11).

##### 3.2.2.2 Subtheme 2: peer connection

Participants also highlighted the interpersonal value of training. Training created opportunities to meet peers from other hospitals and specialties, exchange experiences, and broaden professional horizons. Participants valued these relationships as valuable sources of support and future collaboration. One participant said, “Training can enrich your knowledge and help you meet more people. After I went out for training, I got to know many friends from different hospitals and departments” (P9). Another explained, “Training not only helps us learn a lot of professional knowledge but also allows us to meet colleagues from other places. Compared with places like Beijing, Shanghai, and Guangzhou, we are still behind. Once you get in touch with them, you realize that things we have not even started are already being done very well there. If our department wants to develop these services later, we can still communicate and learn from them” (P13).

##### 3.2.2.3 Subtheme 3: career development

Training was also associated with career development. Participants believed that off-the-job training expanded future possibilities in specialty nursing, case management, continuity of care, chronic disease follow-up, and even management roles. One participant explained, “With the experience of receiving full-time specialty training, there are several directions for development. One is case management, another is long-term chronic disease management, similar to continuity-of-care positions, and another possible direction is community nursing” (P2). Another participant commented, “To some extent, for people who are willing to learn, attending more training should bring more career options later on” (P10). A third said, “Some people, after receiving off-the-job training, can become specialty nurses in their departments. Once they follow a specialized path, they may later move toward management positions” (P11).

#### 3.2.3 Theme 3: competing organizational, personal, and role-related burdens

Although participants clearly recognized the value of training, they also described substantial burdens associated with participation. These burdens arose from departmental realities, performance expectations, and the need to balance multiple family and professional roles. Participation in off-the-job training was therefore often experienced as both an opportunity and a strain.

##### 3.2.3.1 Subtheme 1: departmental constraints

At the organizational level, participants described uneven departmental support for training participation. Staffing shortages, limited training spaces, inconsistent leave arrangements, and differences in departmental policies all affected whether nurses could attend training smoothly. One participant said, “If staffing can be arranged adequately, departments are generally supportive. But when staffing is tight, or during periods such as COVID-19 prevention and control, there are real difficulties in implementation” (P12). Another participant pointed to institutional policy differences: “I think it depends on the policy of each hospital and each department. For example, if I attend a training course and I am not paid, or I even have to use my own leave, then I may be less willing to go” (P10). Limited access to training opportunities was another barrier. As one participant explained, “There are too few places in these training courses, such as those for midline catheters or PICC specialty nursing. I may really want to learn the procedure, but the places are limited, and the department cannot give all the opportunities to one person, so I just have to keep trying” (P14).

##### 3.2.3.2 Subtheme 2: performance pressure

Participants also described the pressure associated with the training process itself. They referred to heavy course loads, strict assessment, and the expectation that training should lead to visible outcomes after returning to work. One participant stated, “After coming back, we still had to report our work—especially in the earlier years, specialty nurses had to report every year on what they had accomplished in the department, but in fact there were not really any major projects to report” (P3). Another participant expressed anxiety about being unable to convert learning into practice: “What I fear most is learning something and then not being able to use it effectively in the department or continue developing it. If the hospital sends you out to learn, the purpose is definitely to bring some benefit back to the department. Especially when training is linked with competitions, the pressure becomes very high. Even though everyone says trying your best is enough, people still hope their efforts will lead to results, and if you do not achieve the result you wanted, it can feel very discouraging” (P8).

##### 3.2.3.3 Subtheme 3: multiple role demands

In addition, many participants described the challenge of balancing training with family responsibilities and other roles outside work. Nurses, especially women with children or limited family support, reported having to navigate competing demands from work, study, caregiving, and everyday life. One participant said, “Now that I am raising a child, this is not just an excuse; it is a real situation. At home, I have both older and younger family members to care for, and these everyday responsibilities can take away some of my energy for learning” (P1). Another explained, “Sometimes the child is still very young, and after going home, I still have to take care of the child. There may also be health issues, work-related factors, and even a family member with cancer who needs care” (P5). A more senior participant reflected on the gendered nature of the nursing workforce: “Our profession is still mainly made up of women. Beyond being a healthcare worker, when I go home, I am also someone's daughter or parent, and I may need to care for children. Family issues have to be balanced as well” (P10). Similarly, one participant said, “Nowadays, many people have two children. If the family support system is not good, then they really may have no way to go for training” (P11).

#### 3.2.4 Theme 4: misalignment in training design, delivery, and feedback

While participants affirmed the intrinsic value of off-the-job training, they expressed dissatisfaction with current pedagogical design and delivery. Reported deficiencies included a lack of systematization, insufficient clinical relevance, rigid formats, and limited mechanisms for feedback or program adjustment.

##### 3.2.4.1 Subtheme 1: limited systematization and practical relevance

One major concern was the lack of systematization and practical applicability in training content. Participants felt that some training programs were too broad, overly theoretical, and insufficiently structured across different competency levels. They also stressed the importance of combining classroom learning with practice-based teaching. One participant said, “Some training is very broad. You learn everything related to oncology, but after finishing it, there is nothing concrete that is really implementable” (P6). Another explained, “These post-employment training programs are relatively general. There is no systematic progression from beginner to intermediate to advanced levels. There is no gradual pathway, and it often feels more like cramming” (P15). A third participant emphasized the gap between theory and practice: “Theory still needs to be combined with on-site teaching. Theory helps you gain knowledge, but for some procedures, if you only explain them, people cannot imagine them and cannot really learn them. They need to do it themselves. If you only tell them or show them, they will never truly learn it” (P8).

##### 3.2.4.2 Subtheme 2: rigid training formats

Participants also viewed current training formats as rigid and insufficiently responsive to learners' schedules and interests. There was a clear preference for increased flexibility, interactive pedagogical approaches, and the integration of online and face-to-face learning. One participant described the current problem this way: “Some face-to-face training courses are very concentrated. The teacher keeps talking, and the learners sit there all day. But in online courses, one teacher finishes and another starts immediately, with almost no time to breathe. Ideally, online and offline formats should be combined so that people have a choice” (P4). Another participant hoped for greater flexibility and choice: “The content could be divided a bit more finely. It does not have to be the same large lecture for everyone every time. I hope there could be elective content, just like at university. First, there are required parts everyone must complete, and then there are elective parts so people can learn what interests them. I also hope online courses can be replayed, rather than becoming unavailable after a few days” (P1).

##### 3.2.4.3 Subtheme 3: inadequate feedback mechanisms

A further concern was the lack of learner assessment and feedback during the training process. Participants wanted training organizers to assess learners' needs before training, communicate with them during training, and adjust content in response to actual learning needs rather than relying only on final examinations. One participant suggested, “Off-the-job training should include asking people what needs they have and what kinds of problems and difficulties they encounter in clinical work. If learning is targeted to these problems and difficulties, it will be more interesting and more useful for the learner” (P5). Another participant stated, “During training, there should be more communication with us, and the teaching content should be adjusted. For example, if everyone already understands something, there is no need to spend too much time on it. Also, not everyone is equally self-disciplined, so there should be some kind of monitoring or prompting mechanism, rather than waiting until the end of training for an exam” (P1).

#### 3.2.5 Theme 5: expectations for post-training application and career pathways

Participants emphasized that the value of off-the-job training depended not only on participation but also on whether newly acquired competencies could be recognized, applied, and connected to visible career opportunities after returning to practice.

##### 3.2.5.1 Subtheme 1: practice application platforms

Many participants wanted their hospitals and departments to provide practical platforms for applying what they had learned. They hoped to have opportunities to share their knowledge, lead relevant clinical activities, and receive the organizational support needed to translate training into practice. One participant explained, “After learning, I think there should be further opportunities to teach others or to lead people in doing this work. That would also make the person who attended training feel more confident. Otherwise, if you come back and there is no result, then the next time I get the chance—even after several years—I may not really want to participate” (P5). Another participant highlighted the structural support required for implementation: “To promote a project, you need funding and time. But in our work, everyone is already filling a fixed post, and there is simply no spare time. Even if you use your own rest time, where will the funding come from? Leadership support is also needed, and you need a team to do this work. One person alone cannot achieve much” (P7). A third participant stressed the importance of institutional platforms: “I think this also requires the department and the hospital to build such a platform and opportunity. Without a platform and opportunity, after learning something, you may not know where to use it, and over time, if you do not use it, what you learned will all be forgotten” (P3).

##### 3.2.5.2 Subtheme 2: career pathways and role recognition

Participants also strongly desired clearer career planning after training. Several said that after investing substantial time and effort in training, they returned to essentially unchanged roles, without matching specialty positions or clearly defined development routes. One participant said, “I really think the hospital needs a clearer career development plan. I have already completed off-the-job training, but after coming back I still do not feel very clear about how I should develop in the future. I want a roadmap, so I know where I am heading, and then I can work with more motivation” (P6). Another participant described the frustration of limited role expansion after training: “After training, I think about how much effort I put in and how many difficulties I overcame. But when I came back, I felt that there was even more work than before, and the department still had no positions beyond that of a clinical nurse. The path for personal development is too narrow. I have really been working hard, but there is no clear direction for that effort” (P2).

Overall, participants suggested that off-the-job training would be more valuable if it were more closely linked to institutional recognition, practice-based application, and visible career progression. Participants did not regard learning alone as adequate. Instead, they emphasized that training outcomes depended critically on whether workplace environments actively supported the translation and sustained use of newly acquired skills into everyday clinical routines.

## 4 Discussion

Guided by sensitizing concepts of training transfer, continuing professional development, organizational support, and specialist-nurse role development, this study demonstrated that oncology nurses did not regard full-time, off-the-job training as an optional educational opportunity (20). Rather, they viewed it as part of what is required to remain clinically current and professionally credible in a specialty characterized by rapidly changing treatments, increasing technical complexity, and expanding expectations of nursing practice. This interpretation is consistent with recent oncology nursing literature, which identifies specialized education and continuing development as essential to safe cancer care and to the maintenance of specialty competence (1, 2, 8).

Participants also described the value of training in broader terms than knowledge acquisition alone. They linked off-the-job training with stronger clinical judgment, greater confidence, wider professional networks, and a clearer sense of future possibility. This finding suggests that training functions not only as continuing education, but also as a form of professional affirmation. Evidence suggests that the value of CPD is inextricably linked to environmental support, which serves as a catalyst for increased nurse engagement and professional autonomy (5). In oncology settings, professional development has also been described as extending beyond educational content to include mentoring, feedback, sponsorship, and career progression (9). Evaluation work in pediatric oncology nursing has likewise shown that continuing education can improve confidence and expand career opportunities in practice (21). Taken together, these findings suggest that nurses are asking not only to be taught, but to be developed.

Despite high motivation, oncology nurses face significant barriers to training participation, including staffing shortages, limited release time, and family obligations. The efficacy of these programs is further challenged by institutional expectations for immediate clinical or professional results. This shifts the interpretation of training barriers away from individual reluctance and toward organizational design. Iwama et al. reported a similar pattern among DNP-prepared nurses, whose contributions were limited by a lack of role clarity, organizational structure, time, and support (22). Consistent with broader evidence from China, excessive workload and limited family support remain significant barriers to training participation. These challenges are often exacerbated by the protracted duration of many specialty programs, which can extend over several weeks, further increasing the cumulative burden on nursing staff (7, 10, 11). Oncology nursing workforce and practice-environment studies have likewise shown that supportive environments are associated with better job satisfaction and stronger intention to stay, whereas inadequate staffing and weak leadership undermine professional well-being (4, 23–25).

Participants were equally clear that the problem was not merely access to training, but the quality and usability of the training they received. Their concern was not simply that there were too few courses, but that training was often too generic, insufficiently staged across levels of competence, and weakly connected to everyday oncology practice. This moves the discussion beyond the familiar claim that more training is needed. What nurses in this study asked for was better-designed training. That position is supported by the oncology education literature, which suggests that programs should be built around clearly identified learner needs rather than broad, undifferentiated curricula 8. Work on early-career oncology professional development in Europe similarly indicates that structured programming, leadership support, and clear educational purpose matter for engagement and perceived value (26). Evidence from a large-scale analysis (*N* = 7,817) indicates a persistent reliance on classroom-based teaching within in-service training. The study also highlights a systemic failure to systematically integrate learner feedback into the assessment of pedagogical outcomes (6). These findings support what our participants described: flexibility, staging, practical application, and timely feedback are central features of effective training design.

An important finding of this study is that participants consistently emphasized what happens after training, particularly whether newly acquired competencies can be applied, recognized, and linked to further professional development. Participants shifted the focus of their evaluation from the immediate learning experience to the tangible utility of the training—specifically, its capacity to create opportunities for practical application, to provide formal recognition of specialty expertise, and to offer clearly defined career advancement. This interpretation is consistent with contemporary oncology nursing research. Professional development in oncology has been described as depending on systems that connect education with recognition, mentoring, role development, and planned advancement (9). Recent research on cancer nursing career frameworks has also emphasized the need for explicit pathways into specialist, advanced practice, education, research, and leadership roles to sustain the workforce (27). Sandeberg et al. 's evaluation of pediatric oncology education similarly concluded that, while training enhanced confidence and career prospects, long-term retention depended on the work environment, work-life balance, and career pathways (21).

Recent Chinese literature consistently indicates a stagnation in career progression for specialty-trained oncology nurses. Participants in previous studies reported minimal changes in job roles and low participation in subsequent academic initiatives, highlighting a systemic lack of formalized post-training integration and longitudinal career support (12). Across several Asian regions, similar gaps in structured oncology nursing education have been identified, and regional initiatives have been developed to strengthen specialist training frameworks (2). Notably, in Hong Kong, oncology nurses may undertake more differentiated professional roles, including chemotherapy nurse and cancer case manager roles (15). This comparison suggests that while specialty roles are inherently viable, their successful implementation depends on formal organizational support and systemic frameworks.

An institutional lens helps make sense of these findings. In a Nursing Outlook study of midwifery student precepting, Alspaugh et al. showed that the most important barriers were not primarily individual, but institutional, located in the state, the clinical setting, and the university (28). Although the context differs from oncology nursing, the analytic relevance is strong. The barriers described by our participants, staffing shortages, leave arrangements, managerial discretion, limited application platforms, and unclear post-training role allocation, were also institutional barriers. They were products of the hospital's organization of work and its valuation of specialty preparation. Evidence suggests that pedagogical improvements must be supported by organizational reform. Without dedicated staffing models and clear pathways for post-training utilization, educational initiatives will fail to achieve the structural specialty capacity required for advanced oncology nursing.

From a nursing management perspective, off-the-job training should be planned as part of oncology workforce development rather than treated as a discrete educational event. Training needs to be tiered across levels of practice, more closely tied to clinical realities, and accompanied by post-training opportunities to teach, lead, implement, and progress. Without those structures, training may still feel personally worthwhile, but its contribution to service development and workforce stability is likely to remain limited. International literature continues to identify pressures on the cancer nursing workforce and emphasizes that specialist education, supportive work environments, and clearer career pathways need to be developed together rather than in isolation (2, 4, 23, 27).

Several limitations should be considered. This single-center study at a Chinese tertiary hospital may reflect site-specific organizational arrangements, necessitating caution when extrapolating the results to other institutional settings. Furthermore, the relatively small sample size may limit the breadth of perspectives captured, and readers should exercise caution when transferring our findings to other contexts. Additionally, male nurses are underrepresented in the sample. Although male participants were not excluded and we actively sought eligible male nurses during purposive sampling, the small pool of male oncology nurses with relevant off-the-job training experience limited recruitment, and gender-related differences in training experiences could not be fully explored.

Participation was limited to those with prior off-the-job training, potentially omitting the viewpoints of nurses lacking such opportunities. Although interviewer familiarity with the clinical setting might have shaped data collection, the lack of hierarchical relationships between researchers and participants reduced potential bias. Another methodological limitation is that we did not adopt a formal theoretical framework for deductively structuring data collection and coding. Although relevant sensitizing concepts supported the interpretation, no pre-existing model was applied to structure the analytic workflow, so the interpretation of themes may still carry a degree of subjectivity, even though our inductive approach allowed the participants' original narratives to take priority. Regardless, the emergent themes of access, workload, and role integration remain highly consistent with wider literature (1, 2, 23).

## 5 Conclusions

Full-time, off-the-job training was perceived by oncology nurses as essential to maintaining specialty competence, broadening professional perspectives, and supporting future career development. At the same time, nurses described substantial burdens related to organizational support, competing work and family responsibilities, and pressure to demonstrate visible post-training outcomes. They also identified unmet needs in training design, flexibility, feedback, post-training application, and career pathway development.

These findings suggest that the value of off-the-job training depends not only on access to educational opportunities but also on whether training is aligned with clinical needs and supported by organizational structures that enable nurses to apply and sustain newly developed competencies in practice. To maximize the impact of specialty nurse training, hospitals should move beyond a training-completion model and establish more learner-responsive, practice-oriented, and organizationally supported systems that connect training with specialty role development, competency utilization, and visible career pathways. Such efforts may strengthen both oncology nursing practice and long-term workforce sustainability.

## Data Availability

The original contributions presented in the study are included in the article/Supplementary Material, further inquiries can be directed to the corresponding author/s.
