# Supplementary material for: Barriers, benefits, and unmet needs in full-time, off-the-job training among oncology nurses: a qualitative descriptive study
Source: Front Med (Lausanne). 2026 Oct 8;13:1979432. doi: 10.3389/fmed.2026.1979432 (PMC13686890; doi:10.3389/fmed.2026.1979432)
Supplement: Supplementary file 1 [file Supplementaryfile1.docx]

Supplementary Material

# 1 Supplementary Tables

## 1.1 Themes, Sub-Themes, and Descriptions

| **Theme** | **Sub-Theme** | **Description** | **Representative codes / condensed meaning units** |
| --- | --- | --- | --- |
| 1. Perceived necessity of off-the-job training for specialty oncology practice | 1.1 Specialty practice demands | Participants viewed off-the-job training as essential rather than optional because oncology nursing required increasingly specialized, professional, and comprehensive practice beyond routine bedside care | High specialty expectations; Need for standardized specialist procedures; Maintaining professional credibility; Patients perceived inconsistency as unprofessional |
|  | 1.2 Clinical and technological updating | Participants regarded off-the-job training as necessary to keep pace with rapidly changing oncology knowledge, guidelines, expert consensus, and specialty procedures | Rapid knowledge updates; Changing guidelines and consensus; Evolving procedures and devices; Avoiding professional stagnation |
| 2. Multidimensional value of training | 2.1 Professional competence | Training was seen as strengthening specialty knowledge, technical skills, clinical reasoning, and evidence-based problem-solving ability in oncology care | Improved theoretical knowledge; Stronger technical skills; Enhanced clinical thinking; Evidence-based problem solving |
|  | 2.2 Peer connection | Training created opportunities to connect with peers from other hospitals, exchange experiences, broaden professional horizons, and build future collaboration networks | Meeting peers; Exchanging experiences; Learning from advanced centers; Building professional networks |
|  | 2.3 Career development | Participants linked training to expanded opportunities in specialty nursing, case management, continuity care, chronic disease follow-up, and management roles | Specialty nurse pathway; Case management opportunities; Broader career options; Possible pathway to management roles |
| 3. Competing organizational, personal, and role-related burdens | 3.1 Departmental constraints | Participation was shaped by staffing shortages, limited training places, inconsistent leave arrangements, and variation in departmental or hospital support | Staffing shortages; Limited training quotas; Difficulty obtaining release time; Use of personal leave; Uneven managerial support |
|  | 3.2 Performance pressure | Training involved heavy coursework, assessment demands, and pressure to demonstrate visible outcomes or achievements after returning to work | Course burden; Assessment pressure; Expectation of outputs; Reporting requirements; Fear of poor results |
|  | 3.3 Multiple role demands | Participants described balancing training with childcare, eldercare, illness, and other family and work responsibilities, especially in a female-dominated workforce | Childcare responsibilities; Eldercare burden; Family illness; Work–family conflict; Limited family support |
| 4. Misalignment in training design, delivery, and feedback | 4.1 Limited systematization and practical relevance | Current training was perceived as overly broad, overly theoretical, and insufficiently staged across beginner-to-advanced levels, with inadequate integration of practice-based teaching | Broad but not implementable content; Overly theoretical teaching; Lack of progressive curriculum; Insufficient hands-on learning |
|  | 4.2 Rigid training formats | Participants wanted more flexible, interactive, and learner-responsive formats, including blended delivery, replayable online content, and greater choice in course structure | Concentrated lecture-based teaching; Inflexible scheduling; Desire for blended learning; Wish for elective content; Replayable online courses |
|  | 4.3 Inadequate feedback mechanisms | Participants reported weak needs assessment, limited communication during training, and insufficient process feedback or content adjustment before final evaluation | Limited pre-training needs assessment; Weak communication during training; Lack of ongoing monitoring; End-point assessment only |
| 5. Expectations for post-training application and career pathways | 5.1 Practice application platforms | Participants emphasized the need for organizational opportunities and platforms to apply, share, and extend what they had learned after training | Opportunities to teach others; Implementing projects after training; Leadership support; Need for team and resources; Lack of institutional platform for application |
|  | 5.2 Career pathways and role recognition | Participants wanted clearer post-training role expectations, formal recognition of specialty capability, and visible pathways for long-term professional development | Unchanged post-training role; Lack of specialty positions; Unclear career roadmap; Limited recognition of capability; Narrow development pathway |

Note. This table was developed from the study’s 5 overarching themes, 13 subthemes, and 135 initial codes. The representative codes/condensed meaning units shown are illustrative rather than exhaustive.

## 1.2 The Consolidated Criteria for Reporting Qualitative Research (COREQ): 32-item checklist

| **No** | **Item** | **Guide questions/description** | **Notes** |
| --- | --- | --- | --- |
| **Domain 1: Research team and reﬂexivity** | | | |
| *Personal characteristics* | | | |
| 1 | Interviewer/  facilitator | Which author/s conducted the interview or focus group? | Two members of our research team conducted the semistructured interviews. |
| 2 | Credentials | What were the researcher’s credentials? *E.g. PhD, MD* | The researchers held a Master of Medicine (MM) degree |
| 3 | Occupation | What was their occupation at the time of the study? | The interviewers’ professional role was nursing; they were nurse researchers involved in this study. |
| 4 | Gender | Was the researcher male or female? | The interviewers were female. |
| 5 | Experience and training | What experience or training did the researcher have? | The interviewers had prior training in qualitative interviewing and prior clinical experience in oncology nursing. |
| *Relationship with participants* | | | |
| 6 | Relationship established | Was a relationship established prior to study commencement? | Yes. The interviewers were acquainted with participants before the interviews, and the team was familiar with the clinical context. |
| 7 | Participant knowledge of the interviewer | What did the participants know about the researcher? *e.g. personal goals, reasons for doing the research* | Participants were informed about the study purpose, procedures, audio recording, confidentiality, and voluntary participation. The manuscript does not further elaborate on what participants knew about the interviewers’ personal motivations. |
| 8 | Interviewer characteristics | What characteristics were reported about the interviewer/facilitator? *e.g. Bias, assumptions, reasons and interests in the research topic* | We reported that the interviewers were familiar with the clinical context, were not in direct managerial roles over participants, and used reflexive discussion to monitor possible assumptions and bias. |
| **Domain 2: Study design** | | | |
| *Theoretical framework* | | | |
| 9 | Methodological orientation and theory | What methodological orientation was stated to underpin the study? *e.g. grounded theory, discourse analysis, ethnography, phenomenology, content analysis* | The study adopted a qualitative descriptive design and used conventional qualitative content analysis. |
| *Participant selection* | | | |
| 10 | Sampling | How were participants selected? *e.g. purposive, convenience, consecutive, snowball* | We used purposive maximum-variation sampling. |
| 11 | Method of approach | How were participants approached? *e.g. face-to-face, telephone, mail, email* | Eligible participants were approached primarily face-to-face in their departments; 1 participant was interviewed by telephone when an in-person interview was not feasible. |
| 12 | Sample size | How many participants were in the study? | Fifteen nurses participated in the study. |
| 13 | Non-participation | How many people refused to participate or dropped out? Reasons? | No invited participants refused to participate or withdrew. When some nurses were unavailable at the initial invitation, interviews were rescheduled. |
| *Setting* | | | |
| 14 | Setting of data collection | Where was the data collected? *e.g. home, clinic, workplace* | Interviews were conducted in a private office in a large tertiary hospital in Shaanxi Province, China; 1 interview was conducted by telephone. |
| 15 | Presence of non-participants | Was anyone else present besides the participants and researchers? | No non-participants were present during the interviews. |
| 16 | Description of sample | What are the important characteristics of the sample? *e.g. demographic data, date* | The sample comprised 15 oncology nurses from 6 oncology-related departments. Thirteen were female and 2 were male; professional titles included senior (n = 3), intermediate (n = 9), and junior (n = 3). Participant characteristics are presented in Table 2. |
| *Data collection* | | | |
| 17 | Interview guide | Were questions, prompts, guides provided by the authors? Was it pilot tested? | Yes. We developed a semistructured interview guide after literature review and team discussion; it was pilot tested and refined before formal data collection. The guide is presented in Table 1. |
| 18 | Repeat interviews | Were repeat interviews carried out? If yes, how many? | Formal repeat interviews were not conducted. However, participants were re-contacted when clarification of specific points was needed during data organization. |
| 19 | Audio/visual recording | Did the research use audio or visual recording to collect the data? | Yes. Interviews were audio-recorded with participants’ consent. |
| 20 | Field notes | Were ﬁeld notes made during and/or after the interview or focus group? | Yes. One interviewer documented nonverbal information, including tone, facial expression, pauses, and emotional reactions. |
| 21 | Duration | What was the duration of the interviews or focus group? | Interviews lasted approximately 30 to 60 minutes; total interview time was 482 minutes. |
| 22 | Data saturation | Was data saturation discussed? | Yes. We reported informational sufficiency and continued sampling until no new themes or subthemes emerged. |
| 23 | Transcripts returned | Were transcripts returned to participants for comment and/or correction? | Full transcripts were not routinely returned. However, participants were re-contacted to verify specific details when clarification was needed. |
| **Domain 3: Analysis and findings** | | | |
| *Data analysis* | | | |
| 24 | Number of data coders | How many data coders coded the data? | Two members of the research team checked transcripts and initial coding against the audio recordings. Theme development was then discussed collaboratively within the research team. |
| 25 | Description of the coding tree | Did authors provide a description of the coding tree? | Yes. A coding tree was provided in Supplementary Table S1, including 5 overarching themes, 13 subthemes, brief descriptions, and representative codes/condensed meaning units. |
| 26 | Derivation of themes | Were themes identified in advance or derived from the data? | Themes were derived inductively from the data through iterative coding, categorization, and theme development. |
| 27 | Software | What software, if applicable, was used to manage the data? | NVivo 11.0 was used to manage and organize the data. |
| 28 | Participant checking | Did participants provide feedback on the findings? | Yes. Preliminary interpretations of the findings were shared with several participants to assess whether the themes were consistent with their experiences. |
| *Reporting* | | | |
| 29 | Quotations presented | Were participant quotations presented to illustrate the themes/ findings? Was each quotation identified? *E.g. participant number* | Yes. Participant quotations were presented throughout the Results section and were identified by participant number (eg, P1, P5, P10). |
| 30 | Data and findings consistent | Was there consistency between the data presented and the findings? | Yes. The reported themes and subthemes were supported by multiple quotations that were consistent with the findings. |
| 31 | Clarity of major themes | Were major themes clearly presented in the findings? | Yes. Five major themes were clearly presented in the Results section. |
| 32 | Clarity of minor themes | Is there a description of diverse cases or discussion of minor themes? | Yes. Thirteen subthemes were presented and illustrated with quotations. The manuscript focused primarily on common patterns, while also reflecting variation across participants where relevant. |
